# Comparative evaluation of microleakage amongst bioactive, hydrophobic and hydrophilic pit and fissure sealants: an *in vitro* study

**DOI:** 10.21142/2523-2754-1402-2026-286

**Published:** 2026-04-04

**Authors:** Ishita Agrawal, Farhin Katge, Manohar Poojari, Shilpa Shetty, Devendra Patil, Palak Nisar

**Affiliations:** 1 Department of Pediatric and Preventive Dentistry, Terna Dental College. Navi-Mumbai, Maharashtra, India. agrawal.ishita310@gmail.com pedotdc@gmail.com manohar_poojari@yahoo.com shilpakarthick@gmail.com dev1987endra@gmail.com palaknisar28@gmail.com Department of Pediatric and Preventive Dentistry Terna Dental College. Navi-Mumbai Maharashtra India agrawal.ishita310@gmail.com pedotdc@gmail.com manohar_poojari@yahoo.com shilpakarthick@gmail.com dev1987endra@gmail.com palaknisar28@gmail.com

**Keywords:** bioactive, dental sealants, fissure sealants, marginal leakage, dental materials, bioactivo, selladores dentales, selladores de fisuras, filtración marginal, materiales dentales

## Abstract

**Background:**

Pit and fissure sealants are widely used to prevent occlusal caries, but their effectiveness depends on adequate marginal sealing. Newer sealants with hydrophilic and bioactive properties have been introduced to enhance performance. The present study aimed to evaluate and compare the microleakage of bioactive, hydrophobic, and hydrophilic pit and fissure sealants in premolars. The aim of the present study was to evaluate and thereby compare the microleakage using bioactive, hydrophobic and hydrophilic pit and fissure sealant in premolars.

**Materials and Methods:**

Forty-five extracted human premolars were randomly allocated into three groups of 15 each. Group A comprise of Helioseal F; Group B: UltraSeal XT® hydro ^TM^ and Group C: Bioactive BioCoat ^TM^. Further, the teeth were subjected to thermocycling and immersed in 0.2% Rhodamine-B dye solution for a day. Buccolingual sectioning of teeth was done and evaluated under stereomicroscope. The mean score was subjected to one-way analysis of variance (ANOVA) to assess inter-group differences.

**Result:**

Highest microleakage was seen in group A (1.13 ± 1.18) followed by group B (0.60 ± 0.91) and group C (0.46 ± 0.51). One way- analysis of variance was carried out and it was observed that no statistically significant difference was seen in all three groups (p = 0.119).

**Conclusion:**

All three pit and fissure sealants demonstrated microleakage, with the bioactive sealant showing the least and the hydrophobic sealant the highest values.

## RESUMEN

**Antecedentes:**

Los selladores de fosas y fisuras se utilizan ampliamente para prevenir la caries oclusal, pero su eficacia depende de un sellado marginal adecuado. Se han introducido nuevos selladores con propiedades hidrofílicas y bioactivas para mejorar su rendimiento. El presente estudio tuvo como objetivo evaluar y comparar la microfiltración de selladores de fosas y fisuras bioactivos, hidrofóbicos e hidrofílicos en premolares.

**Materiales y métodos:**

Cuarenta y cinco premolares humanos extraídos se asignaron aleatoriamente a tres grupos de 15 cada uno. El grupo A recibió Helioseal F; el grupo B, UltraSeal XT® hydro™; y el grupo C, Bioactive BioCoat™. Posteriormente, los dientes se sometieron a termociclado y se sumergieron en una solución de rodamina B al 0,2 % durante un día. Se realizó un corte bucolingual de los dientes y se evaluó con un estereomicroscopio. La puntuación media se sometió a un análisis de varianza (ANOVA) de un factor para evaluar las diferencias entre los grupos.

**Resultado:**

La mayor microfiltración se observó en el grupo A (1,13 ± 1,18), seguido del grupo B (0,60 ± 0,91) y el grupo C (0,46 ± 0,51). El análisis de varianza de un factor no mostró diferencias estadísticamente significativas entre los tres grupos (p = 0,119).

**Conclusión:**

Los tres selladores de fosas y fisuras presentaron microfiltración; el sellador bioactivo mostró los valores más bajos y el sellador hidrófobo los más altos.

## INTRODUCTION

Occlusal pit and fissures constitute one of the most caries-prone sites in the dentition due to their complex and retentive anatomy, which favors the entrapment of food debris and microbial colonization ^1^. Conventional oral hygiene methods, including toothbrushing, often fail to effectively cleanse these deep fissures, thereby predisposing them to the initiation of dental caries. With the increasing acceptance of minimal intervention dentistry, the emphasis has shifted from invasive restorative approaches to preventive strategies that aim to preserve tooth structure and control the disease process ^2^. Among these strategies, the use of pit and fissure sealants has emerged as a highly effective measure for the prevention of occlusal caries. Sealants act as a physical barrier between acidogenic microorganisms and susceptible fissures, thereby preventing demineralization and subsequent lesion formation. Furthermore, their application has been associated with a reduction in treatment costs when compared with restorative procedures, and they also contribute to a decreased incidence of new carious lesions ^3^^-^^5^. Long-term follow-up studies provide moderate evidence indicating that individuals treated with sealants exhibit significantly fewer carious lesions on occlusal surfaces of permanent molars compared to untreated individuals, even after a period exceeding seven years ^6^.

The majority of commercially available pit and fissure sealants are resin-based and hydrophobic in nature. Their retention is achieved through micromechanical interlocking with etched enamel surfaces ^7^. However, the clinical effectiveness of these sealants may be compromised under suboptimal conditions, particularly in pediatric dentistry, where isolation is challenging and moisture contamination is common. Moisture adversely affects the adhesion at the sealant-enamel interface, ultimately leading to reduced retention and an increased risk of microleakage ^8^. To overcome these limitations, hydrophilic resin-based sealants have been introduced. Their moisture-tolerant nature allows for more reliable adhesion under clinical conditions where absolute isolation is difficult to achieve, thus enhancing their preventive efficacy ^9^. Certain hydrophilic sealants are formulated with a high proportion of radiopaque filler particles to improve mechanical strength and exhibit fluorescent properties, which facilitate easy identification of material retention during follow-up examinations ^10^.

More recently, advancements in dental biomaterials have led to the development of bioactive resin-based sealants. These materials incorporate novel technologies such as semi-permeable resin microcapsules, which encapsulate and gradually release fluoride, calcium, and phosphate ions into the surrounding environment ^11^. The release and recharge of these ions create a locally saturated environment that promotes remineralization of enamel and increases resistance to acid challenges ^12^. Such bioactivity offers the potential dual benefit of sealing pits and fissures while simultaneously enhancing the tooth’s natural defense mechanisms.

The clinical success of pit and fissure sealants depends not only on their retention but also on their ability to provide an effective marginal seal. Inadequate sealing permits microleakage, which allows penetration of bacteria, oral fluids, and ions into the interface, creating conditions conducive to the initiation and progression of secondary caries beneath the sealant ^13^. Thus, evaluating the microleakage properties of conventional hydrophobic, newer hydrophilic, and bioactive sealants is essential in determining their relative effectiveness and longevity in preventive dentistry. 

For the present study, the null hypothesis stated that there is no difference in the microleakage exhibited by hydrophobic, hydrophilic, and bioactive pit and fissure sealants, whereas the alternative hypothesis stated that a difference exists in the microleakage exhibited by these three types of sealants.

## MATERIALS AND METHODS

The study was approved from the Institutional Review Board (IRB) - Ethics Committee (TDC- IEC-TDC/42/2020). Using G* power software (version 3.0.10), sample size was calculated in accordance with the findings of Butail *et al*. ^14^. Forty-five extracted sound maxillary and mandibular premolars extracted for orthodontic purposes or periodontal pathology. Teeth with intact deep pit and fissures were included in the study. Teeth with obvious dental caries, temporary or permanent restoration, worn out occlusal surface due to attrition, abrasion or erosion and teeth with any visible developmental disturbances like hypoplasia, hypo mineralisation were excluded from the study.

### Sample preparation

**Figure 1:**
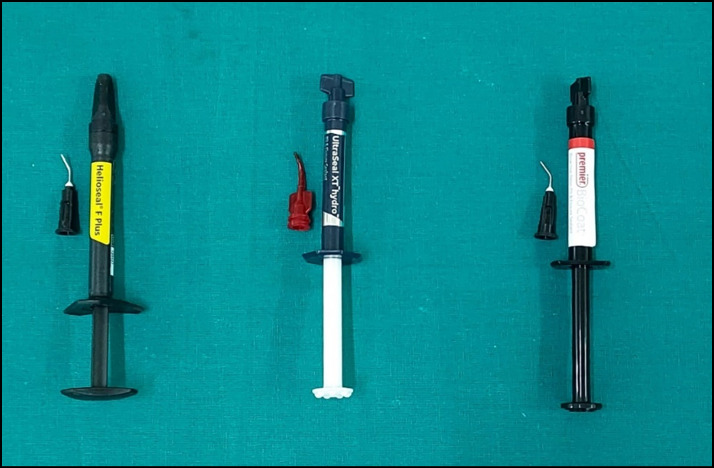
Pit and fissure sealants used for all the three groups

The extracted teeth were collected from different dental institutions and private clinics and were stored in 0.01% thymol before use. Extracted teeth were cleaned with aqueous pumice slurry and prophylactic bristle brush prior to the procedure with slow-speed contra-angle hand piece. The extracted teeth were kept in 0.9% isotonic saline at room temperature till further use to prevent teeth from dehydration. The extracted teeth were randomly allocated into three groups of 15 each. Group A was treated with a hydrophobic resin-based sealant (Helioseal F Plus, Ivoclar Vivadent, Schaan, Liechtenstein). Group B received a hydrophilic resin-based sealant (UltraSeal XT® hydro™, Ultradent, South Jordan, USA). Group C was treated with a bioactive resin-based sealant (BioCoat™, Premier Dental Products, Plymouth Meeting, PA, USA). (Figure 1) The detailed composition and properties of the hydrophobic, hydrophilic, and bioactive sealants employed in this study are presented in Table 1.

**Table 1:** Composition and properties of pit and fissure sealants used in the study

Group	Material (Trade name)	Type	Main components	Manufacturer
A	Helioseal F	Hydrophobic resin-based sealant	Bis-GMA*, TEGDMA*, fluoride-releasing fillers	Ivoclar Vivadent, Liechtenstein
B	UltraSeal XT® hydro™	Hydrophilic resin-based sealant	Urethane dimethacrylate, methacrylated phosphoric ester, 53 wt.% radiopaque inorganic filler, fluoride	Ultradent Products Inc., USA
C	BioCoat™	Bioactive resin-based sealant	Resin matrix with SmartCap™ microcapsules containing fluoride, calcium and phosphate ions	Premier Dental Products, USA

Bis-GMA* = Bisphenol A-glycidyl methacrylate, TEGDMA*sss = Triethylene glycol dimethacrylate

### Sealant Application

Using 37% phosphoric acid (Ivoclar Vivadent, Liechtenstein) occlusal surface was etched for 20 seconds for all the three groups. The teeth were gently rinsed with water for 20 second followed by air drying till frosty appearance was visible. If frosty appearance was not visible re-etching was performed. Sealant application was completed according to manufacturer’s instruction in all three groups. In order to avoid air entrapment and voids, the tip of explorer no 17/23 was gently moved through the occlusal surface. Polymerization was carried out for 20 seconds under LED curing light (Woodpecker, China). Following the application of sealants, teeth were kept in distilled water at room temperature for 24 hours. 500 cycles between 5 °C and 55 °C of thermocycling was performed with each bath requiring 30 seconds of immersion time and 10 seconds of transfer time.

### Preparation of sample for microleakage assessment

To test the effectiveness of pit and fissure sealants for microleakage, samples were further assessed. All the apices of teeth were sealed with sticky wax. Except for a 2 mm wide zone surrounding the sealant borders, the occlusal surface was coated twice with nail varnish to limit dye penetration to the sealant margin. The teeth were stored in deionized water once the varnish dried to prevent dehydration. To allow dye penetration along the margins of the pit and fissure sealant, teeth were immersed in solution of 0.2% Rhodamine-B dye for a day. To eliminate any extra dye, teeth were thoroughly cleaned and dried. A low speed water cooled diamond disc was used to cut each tooth into two sections bucco-lingually through the sealant. The resulting specimens were observed under a stereomicroscope at a 40X magnification to check for microleakage. All images were saved in jpeg format. Dye penetration was graded based on 4-point scoring system by two trained observers using Ovrebo and Raadal ^15^ criteria for evaluating dye penetration.

### Scoring assessment

**Figure 2:**
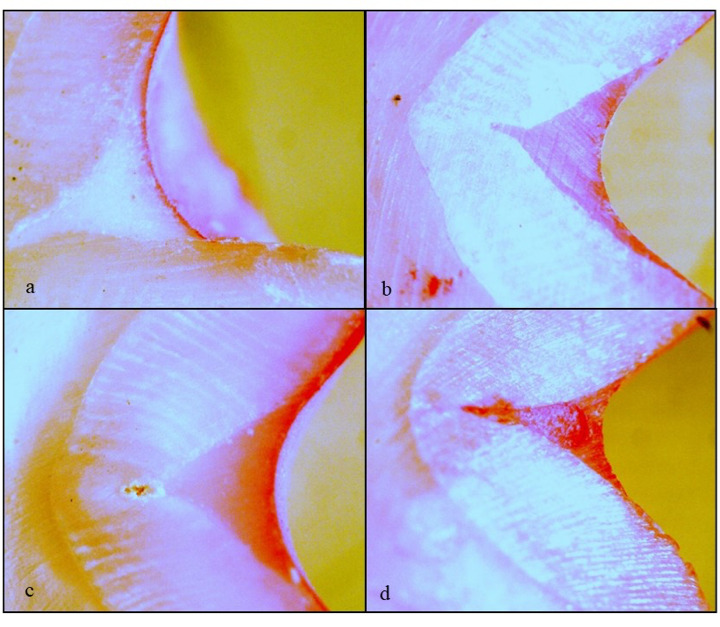
a) Microleakage score 0-no dye penetration; b) Microleakage score 1-dye penetration in the outer half of the fissure; c) Microleakage score 2-dye penetration in the inner half of the fissure; d) Microleakage score 3-dye penetration to the base of the fissure.

Scoring criteria for microleakage assessment was done as Score 0: No dye penetration, Score 1: Dye penetration restricted to outer half of enamel- sealant interface, Score 2: Dye penetration in inner half of enamel-sealant interface and Score 3: Dye penetration into underlying fissure (Figure. 2)

### Statistical analysis

Prior to statistical analysis, the distribution of microleakage scores in each group was assessed to ensure conformity with the assumptions of parametric testing. The descriptive statistics indicated notable variability, particularly in Group A, where the standard deviation was comparable to the mean, suggesting heterogeneity within the group. Normality of the data was evaluated using the Shapiro-Wilk test, which confirmed that the distribution of microleakage scores did not significantly deviate from normality in any of the groups.

The collected data were imported into the Statistical Package for the Social Sciences (SPSS) for Windows, version 16.0 (SPSS Inc., Chicago, IL, USA). Descriptive statistics, including mean and standard deviation, were calculated to summarize the characteristics of the sample. Percentage distribution of microleakage scores was determined using the Chi-square test. To evaluate differences in microleakage between groups, the scores were subjected to one-way analysis of variance (ANOVA). All tests were two-sided, and a significance level of p < 0.05 was applied.

## RESULTS

**Figure 3:**
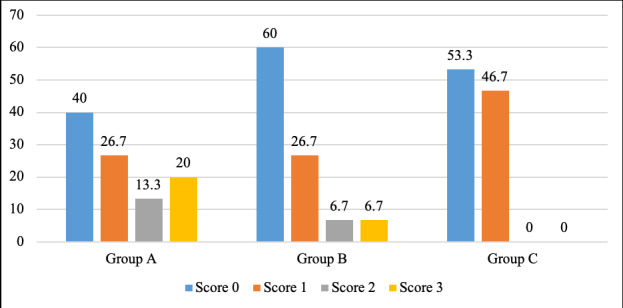
Bar graph depicting percentage of samples of all three groups showing microleakage score

Microleakage was observed in all three pit and fissure sealant groups. The hydrophobic resin-based sealant (Group A, Helioseal F Plus) exhibited a trend toward higher microleakage, whereas the bioactive resin-based sealant (Group C, BioCoat™) showed lower microleakage and more consistent performance across specimens (Table 2, Figure 3). Data distribution was confirmed to be normal using the Shapiro-Wilk test, allowing parametric analysis. One-way ANOVA indicated no statistically significant differences in microleakage among the three sealant types (p = 0.119) (Table 2).

**Table 2:** Mean microleakage scores in three different groups

ANOVA test				
Group	N	Mean	SD	P
A	15	1.13	1.19	0.119
B	15	0.60	0.91	
C	15	0.47	0.52	

**Figure 4:**
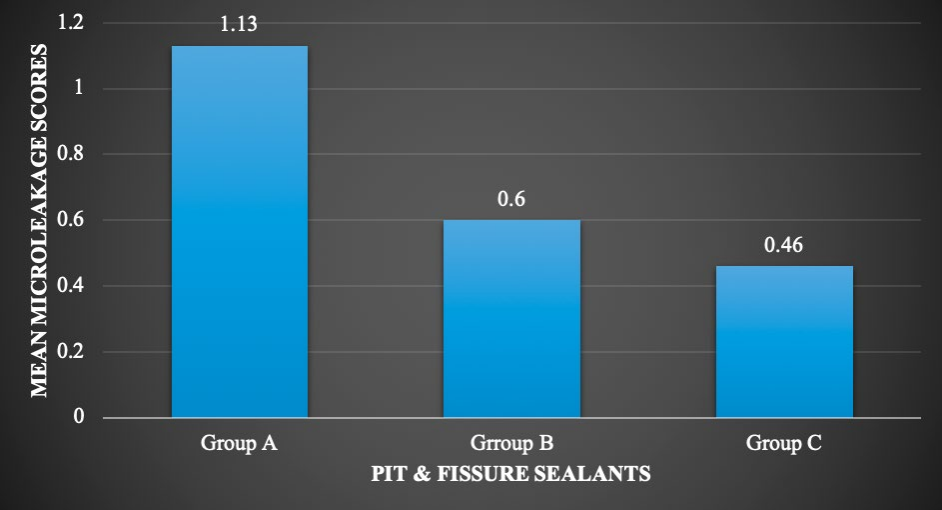
Bar graph depicting mean microleakage scores of samples of all three groups

The hydrophilic sealant (Group B, UltraSeal XT® hydro™) demonstrated intermediate performance. The percentage distribution for microleakage score 0, 1, 2 and 3 was found to be 40%, 26.7%, 13.3% and 20 % for group A. The percentage distribution for microleakage score 0,1, 2 and 3 was found to be 60%, 26.7%, 6.7% and 6.7% for group B. The percentage distribution for microleakage score 0 and 1 was 53.3% and 46.7% respectively for group C with no score of 3 and 4. The mean microleakage score (Figure 4) for group A (Helioseal F Plus) was found to be greater; 1.13 ± 1.18 followed by group B (UltraSeal XT Hydro) and group C (BioCoat) which was 0.60 ± 0.91 and 0.46 ± 0.51 respectively. (Figure 3)

Figure 4 presents a bar graph of mean microleakage scores with standard deviations, visually highlighting the trend of reduced microleakage in the bioactive sealant group and higher variability in the hydrophobic sealant group. These findings suggest that, although minor differences exist, all three sealant types provide comparable marginal sealing under the conditions of this study.

## DISCUSSION

Pit and fissure sealants are an effective addition to other preventive measures for occlusal caries ^16^. Pit and fissure sealants are described by Simonsen *et al*. ^17^ as a substance that is placed inside the occlusal pit and fissures of teeth that are prone to dental caries, providing a micromechanically bonded protective layer that prevents the caries-causing bacteria from accessing their source of nutrients According to Ganesh and Shobha ^18^, the marginal adaptation of a sealant with enamel surface creates tight seal and minimises microleakage, is the main element impacting its performance and longevity. Therefore, present study was designed to evaluate and compare the microleakage using bioactive, hydrophobic and hydrophilic pit and fissure sealant. Ansari *et al*. ^19^ stated that the most widely accepted technique to clean the tooth surface is use of pumice slurry at slow speed hand piece to remove debris from the enamel surface. This also helps to improve sealant retention by removing plaque from the surface of the enamel, which in turn lowers the risk of microleakage. For cleaning the occlusal surfaces of premolars, prophylaxis was carried out using pumice slurry. The nature of the sealants may be affected by invasive technique like enameloplasty, which were not used in this investigation, as stated by Prabhakar *et al*. ^20^.

The recommended etching time for permanent tooth as per International Association for Dental Research (IADR) sealant symposium (1991) is 20 seconds. In the present study, 37% phosphoric acid was used for 20 seconds as an etchant in all three groups to enhance sealant bonding. The use of an etchant creates honeycomb like structure that enhances surface energy and also accelerates penetration of sealant ^21^. According to published literature sealant placement can be done with or without bonding agent which is specific for individual sealants.

According to Boksman *et al*. ^22^, using a bonding agent does not improve sealant retention. For sealant retention, complete etchant penetration into pits and fissures is necessary ^23^. According to Pinar *et al*. ^24^, who evaluated the clinical effectiveness of fissure sealants both with and without bonding agent, and found that the clinical success of the sealant was unaffected by the bonding agent. Published literature shows no difference in the retention of sealants when applied with or without bonding agent.

Thermocycling is one of the frequently used techniques to reproduce intra oral thermal changes in an in vitro study. Similar to investigations by Penugonda *et al*. ^25^ and Sytner D *et al*. ^26^, samples in the current study were subjected to 500 heat cycles between 5 °C and 55 °C with a dwell period of 30 seconds. Additionally, in order to simulate the oral environment, the marginal microleakage test should be preceded by thermocycling in water bath temperatures between 5 °C and 55 °C, as specified by ISO regulation (ISO TR 11450) ^27^.

The extent of dye penetration was evaluated at a magnification of 40 X under stereomicroscope. The findings of the current study revealed that all three groups had some dye penetration. This result is consistent with findings made by Theodoridou-Pahini *et al*. ^28^ and de Araujo *et al*. ^29^ who claimed that microleakage is a possibility with all restorative materials. This could be due to the fact that sealants coefficient of thermal expansion is significantly higher than the enamel's coefficient of thermal expansion.

Group A showed microleakage score 3 in 20% of the samples tested. On the other hand, in group C none of the sample had exhibited microleakage score of 2 and 3. Similar results were reported by Prabhakar J *et al*. ^30^ where the level of microleakage was greater than that of observed in hydrophobic group (Clinpro) as compared to hydrophilic (Ultraseal XT hydro). 

In a study conducted by Prabhakar J *et al*. ^31^ where they stated that Clinpro, a hydrophobic sealant, had a higher viscosity than UltraSeal XT Hydro. Similar effects were discussed by Irinoda *et al*. ^32^ in their study, which revealed that a higher sealant viscosity may result in poor adaptability and limited penetration into pits and fissures, which would limit retention. Low-viscosity sealants have a better chance of flowing, covering the surface more quickly, and penetrating. However, Barnes *et al*. ^33^ have shown that the viscosity and flow properties of the fissure sealants have no effect on their ability to seal. The current study showed that group B and C (UltraSeal XT Hydro & Biocoat) sealants had lower mean microleakage scores than group A (Helioseal F Plus) sealants. This might be because of the following reasons attributed to firstly, thixotropic characteristic of BioCoat & UltraSeal XT Hydro, which can adhere to the slightly moist teeth generating an impermeable sealant enamel contact. Second, the group II adhesive technology (UltraSeal XT Hydro) produces stronger bonds. Hence, lower microleakage and greater retention are produced by higher bond strength. Finally, when resin-based sealant (Helioseal F Plus) is applied, contaminations from moisture or wetness have a negative impact on marginal sealing ability.

The findings of this study are consistent with those of Prasanna Kumar *et al*. ^34^ and Nahid *et al*. ^35^ who demonstrate improved retention and sealability of hydrophilic sealant. The results of the present investigation, however, were at odds with those of Eliades *et al*. ^36^ who found that sealants made with hydrophobic monomers had better sealing properties than those made with hydrophilic monomers. 

Biocoat has shown some promising results in other studies too. In a recent study that compared the flexural strength, elastic modulus, and remineralization capacities of bioactive and non-bioactive sealant materials after seven days of pH cycling, it was found that BioCoat had the highest concentrations of fluoride, calcium, and phosphate ions and the smallest demineralization area ^37^. In addition, they outperformed non-bioactive pit and fissure sealants in terms of their flexural strength and elastic modulus. AlQahtani *et al*. ^38^ reported about micro tensile bond strength of bio-resin based sealant in aged specimens which was superior to resin based sealant. This might be explained by the fact that both the enamel and bio -Resin based sealant substance include calcium and phosphate ions.

Salma *et al*. ^39^ assessed the effects of bioactive and fluoride fissure sealants on the calcium and phosphate content as well as the surface topography of artificially demineralized enamel in young permanent teeth. By accelerating the biological process of remineralization, adding bioactive material through microcapsules to pit and fissure sealant produced superior outcomes than adding fluoride.

In present study, bioactive sealant showed better adaptability than hydrophobic and hydrophilic sealants. Considering the limitations of the study, Bioactive sealants have shown to be a promising material in prevention of pit and fissure caries. 

One of the limitations of this study was that, it was an in vitro examination and moisture control was easily accomplished. Sealants may function differently depending on the environment due to a variety of circumstances including the type of fissure, isolation, etching, and contamination of fissures. To clarify the precise clinical contribution, additional in vivo investigations, additional pit and fissure sealants, and different preparation techniques should be conducted. Based on the limitation of current study, more clinical trials are required to establish firm findings about the microleakage of different pit and fissure sealants because the clinical outcome of any sealant cannot be anticipated only on the basis of an in vitro study.

## CONCLUSION

Based on the findings of the present study, it can be concluded that all pit and fissure sealants tested exhibited some amount of microleakage. Bioactive BioCoat showed a minimum level of microleakage as compared to hydrophobic, hydrophilic pit and fissure sealant. Thus it can be used as an alternative to conventional resin based pit and fissure sealants.
